# Risk factors for acute kidney injury after liver transplantation in intensive care unit: a retrospective cohort study

**DOI:** 10.1590/1516-3180.2021.0641.R2.12112021

**Published:** 2022-08-08

**Authors:** Ana Paula Camargos de Figueirêdo Neves, Angélica Gomides dos Reis Gomes, Paula Frizera Vassallo, Ana Cristina Simões e Silva, Francisco Guilherme Cancela e Penna, Fabrício de Lima Bastos, Mateus Rocha Muniz, Guilherme Carvalho Rocha, Augusto Cesar Soares dos Santos, Cecilia Gómez Ravetti, Vandack Nobre

**Affiliations:** I MD. Physician and Master’s Student, Postgraduate Program on Infectology and Tropical Medicine, Universidade Federal de Minas Gerais (UFMG), Belo Horizonte (MG), Brazil.; II MD. Physician and Master’s Student, Postgraduate Program on Infectology and Tropical Medicine, Universidade Federal de Minas Gerais (UFMG), Belo Horizonte (MG), Brazil.; III MD, PhD. Physician in the Intensive Care Unit, Hospital das Clínicas, Universidade Federal de Minas Gerais (UFMG), Belo Horizonte (MG), Brazil.; IV MD, PhD. Physician, Department of Pediatrics, Universidade Federal de Minas Gerais (UFMG), Belo Horizonte (MG), Brazil.; V MD, PhD. Physician, Instituto Alfa de Gastroenterologia (IAG), Universidade Federal de Minas Gerais (UFMG), Belo Horizonte (MG), Brazil.; VI MD. Physician, Department of Internal Medicine, School of Medicine, Universidade Federal de Minas Gerais (UFMG), Belo Horizonte (MG), Brazil.; VII Undergraduate Medical Student, Universidade Federal de Minas Gerais (UFMG), Belo Horizonte (MG), Brazil.; VIII Undergraduate Medical Student, Universidade Federal de Minas Gerais (UFMG), Belo Horizonte (MG), Brazil.; IX MD, PhD. Physician, Department of Nephrology, Hospital das Clínicas, Universidade Federal de Minas Gerais (UFMG), Belo Horizonte (MG), Brazil.; X MD, PhD. Physician, Department of Internal Medicine, Universidade Federal de Minas Gerais (UFMG), Belo Horizonte (MG), Brazil.; XI MD, PhD. Physician, Postgraduate Program on Infectology and Tropical Medicine, Universidade Federal de Minas Gerais (UFMG), Belo Horizonte (MG), Brazil.

**Keywords:** Intensive care units, Risk factors, Mortality, Acute renal injury, Acute kidney injury, Liver transplantation, Clinical outcome, Postoperative, Critically ill patients

## Abstract

**BACKGROUND:**

Acute kidney injury (AKI) is a frequent complication during the postoperative period following liver transplantation. Occurrence of AKI in intensive care unit (ICU) patients is associated with increased mortality and higher costs.

**OBJECTIVE:**

To evaluate occurrences of moderate or severe AKI among patients admitted to the ICU after liver transplantation and investigate characteristics associated with this complication.

**DESIGN AND SETTING:**

Single-center retrospective cohort study in a public hospital, Belo Horizonte, Brazil.

**METHODS:**

Forty-nine patients admitted to the ICU between January 2015 and April 2017 were included. AKI was defined from a modified Kidney Disease Improving Global Outcomes (KDIGO) score (i.e. based exclusively on serum creatinine levels).

**RESULTS:**

Eighteen patients (36.7%) developed AKI KDIGO 2 or 3; mostly KDIGO 3 (16 out of the 18 patients). Lactate level within the first six hours after ICU admission (odds ratio, OR: 1.3; 95% confidence interval, CI: 1.021-1.717; P = 0.034) and blood transfusion requirement within the first week following transplantation (OR: 8.4; 95% CI: 1.687-41.824; P = 0.009) were independently associated with development of AKI. Patients with AKI KDIGO 2 or 3 underwent more renal replacement therapy (72.2% versus 3.2%; P < 0.01), had longer hospital stay (20 days versus 15 days; P = 0.001), higher in-hospital mortality (44.4% versus 6.5%; P < 0.01) and higher mortality rate after one year (44.4% versus 9.7%; P = 0.01).

**CONCLUSION:**

Need for blood transfusion during ICU stay and hyperlactatemia within the first six postoperative hours after liver transplantation are independently associated with moderate or severe AKI. Developing AKI is apparently associated with poor outcomes.

## INTRODUCTION

Acute kidney injury (AKI) is a frequent complication during the postoperative period following liver transplantation, with consequent increases in hospital stay, deaths and costs.^1,2^ Moreover, it is associated with an increased risk of developing chronic kidney disease,^1–3^ acute graft failure,^1,3^ sepsis and coagulopathy.^4^ The incidence of AKI during the immediate postoperative period following liver transplantation ranges from 17% to 95% in different series,^1,5,6^ and 8% to 17% of these patients require renal replacement therapy (RRT).^4^ In Brazil, the reported incidence of AKI after liver transplantation has ranged from 32% to 72%, according to the definition criteria used; and the 30-day mortality in this group of patients has ranged from 12% to 25%, but may reach 50% among those requiring RRT.^7–10^

Liver transplant recipients’ survival has improved substantially over the last decades. Nevertheless, occurrences of AKI in this population remain correlated with elevated mortality during the postoperative period. Additionally, even successful liver transplant patients seem to be at higher risk of chronic kidney disease, seen at the long-term follow-up, when they developed AKI during the first postoperative days.^11,12^ The pathophysiology of AKI in these cases is multifactorial.^9,13^ The most likely contributing factors include higher occurrence of hepatic ischemia-reperfusion injury (HIRI),^10,11^ increased use of marginal or high-risk grafts and presence of receptors with a high Model of End-Stage Liver Disease (MELD) score.^12,14^

Currently, Brazil is the country with the largest absolute number of liver transplantations in Latin America and the third globally, with more than 1,700 surgeries per year.^15^ However, few studies in this country or in other low to middle-income countries have assessed AKI during the postoperative period following liver transplantation.

## OBJECTIVE

Given this scenario, the aim of this study was to evaluate occurrences of moderate or severe AKI among patients undergoing liver transplantation at a reference center in Brazil and to investigate associated factors and consequences of this complication.

## METHODS

### Study design and setting

A retrospective cohort study was conducted among adult patients (aged 18 years or older) who had undergone orthotopic cadaveric liver transplantation. These patients were admitted to an intensive care unit (ICU) during their immediate postoperative period (IPO), between January 2015 and April 2017. The exclusion criteria were the occurrence of dialytic chronic end-stage renal failure, double transplantation (hepatic-renal; creatinine clearance (CrCl < 30 ml/minutes) and death during the intraoperative period or within the first 24 hours after ICU admission. The adult ICU of Hospital das Clínicas, Universidade Federal de Minas Gerais (HC-UFMG), is a reference center for liver transplantation, with a total of 18 beds.

This study was approved by the local Research Ethics Committee (CAAE: 83336918.2.0000.5149), on October 8, 2018, which waived the requirement to obtain a signed consent form from the participants, due to the retrospective nature of this study.

### Clinical outcomes

We assessed occurrences of AKI during the first seven days of follow-up or up to the time of hospital discharge or death, whichever came first. We also investigated associations between AKI and some relevant patient outcomes, namely length of ICU and hospital stay, in-hospital mortality and mortality rate after one year.

### Data collection

Data were extracted from the electronic medical records of Hospital das Clínicas, Universidade Federal de Minas Gerais (HC-UFMG), and from the Alfa Institute of Gastroenterology platform (Belo Horizonte, Brazil).^16^ The demographic and clinical characteristics of all participants were obtained. The baseline characteristics assessed were age, gender, body mass index (BMI), Sequential Organ Failure Assessment (SOFA)^17^ score values, presence of comorbidities, reason for liver transplantation, Model for End-stage Liver Disease (MELD)^18^ values, Child-Pugh score values^19^ and laboratory data, including hemoglobin, creatinine (pre-transplantation and post-transplantation) and serum lactate levels. Surgical and cold ischemia time, vasopressors and inotropic medication requirement, along with blood component transfusion, were assessed as intraoperative variables. We also assessed data regarding ICU stay, need for surgical reintervention, need for vasopressors and inotropic drugs and renal replacement therapy rate. Donor-related data, including age, gender, use of vasopressors and occurrence of cardiac arrest, were also collected.

AKI was defined as a 1.5-fold increase in baseline serum creatinine (SCr) level during the first seven days or an increase in SCr ≥ 0.3 mg/dl within 48 hours following liver transplantation. We used a modified AKI KDIGO score (without diuresis measurement) to classify the stage of AKI as KDIGO 1, 2 or 3.^20^

### Statistical analysis

Categorical variables were expressed as absolute and relative frequencies and were compared using the chi-square test or Fisher’s exact test, as indicated. Continuous nonparametric variables were expressed as medians and interquartile ranges (Q1-Q3) and were compared using the Mann-Whitney test.

Patients with AKI were stratified into the subgroups 1, 2 and 3, in accordance with their AKI KDIGO score. Thus, they were classified into two groups: non-AKI and AKI KDIGO 1 (absent or mild) or AKI KDIGO 2/3 (moderate to severe), for further comparative analyses.

Variables with P-value < 0.20 were included in a multivariate analysis (logistic regression model) in order to determine which of the patients’ characteristics were independently associated with development of AKI (KDIGO 2 or 3).

A bicaudal P-value < 0.05 was used to determine significance in all analyses. The SPSS 22.0 software, version 20.0 (IBM, New York, United States), was used for data analysis.

## RESULTS

**Figure 1:**
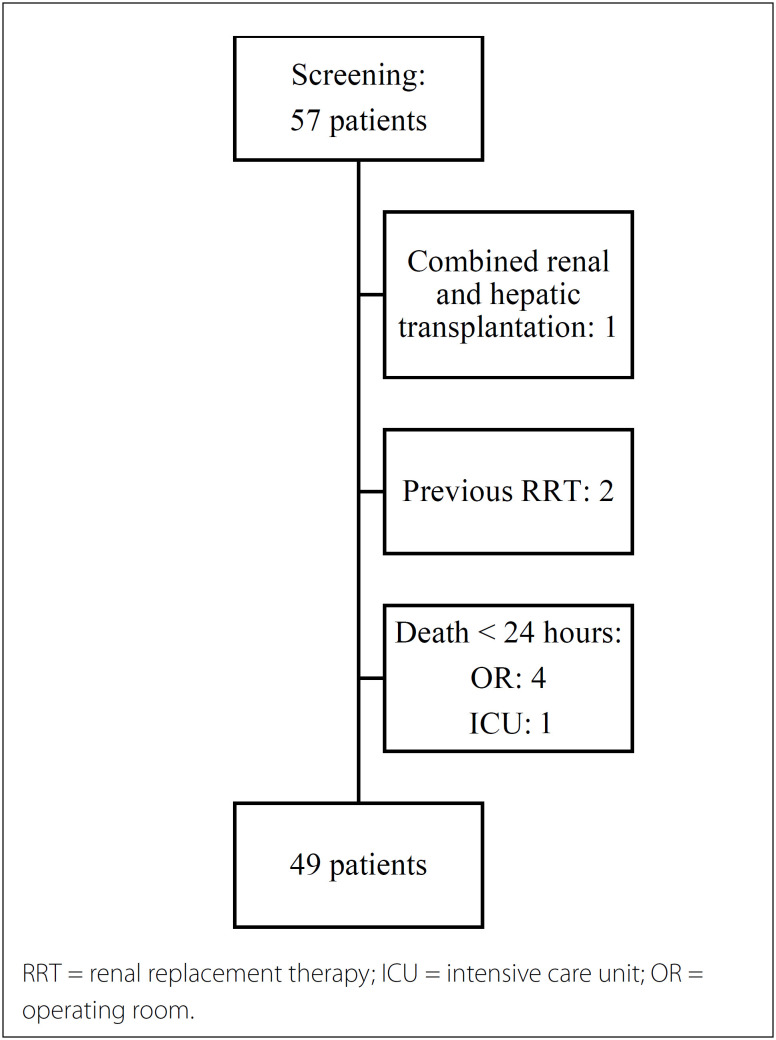
Flowchart of study selection.

Out of a total of 57 patients who underwent liver transplantation during the study period, 49 were included in the final analyses (Figure 1).

The main baseline characteristics of the patients included are shown in Table 1. The median (Q1-Q3) age was 54 years (43-65), and 30 patients (61.2%) were male. The median total duration of the surgical procedure was 407 (309-549) minutes. The median cold ischemia time was 466 (405-633) minutes.

**Table 1:** Main characteristics of the patients included in the study, stratified according to the occurrence of acute kidney injury (AKI) KDIGO 0/1 (no AKI or KDIGO 1) or KDIGO 2 or 3

		All (n = 49)	KDIGO 0/1 (n = 31)	KDIGO 2/3 (n = 18)	P-value
**Preoperative data**					
	Age in years	54 (43-65)	53 (41-62)	61 (43-65)	0.24
	Sex, male	30 (61.2)	20 (64.5)	10 (55.6)	0.55
	Weight in kg	74 (63-82)	72 (63-80)	79.5 (61-86)	0.23
**Comorbidities**					
	SAH	9 (18.4)	6 (19.4)	3 (16.7)	1
	DM	9 (18.4)	4 (12.9)	5 (27.8)	0.25
	CKD	2 (4.1)	2 (6.5)	0	0.52
	Others	21 (42.9)	12 (38.7)	9 (50)	0.55
**Reason for transplantation**					0.42
	HCC	12 (24.5)	5 (16.1)	7 (38.9)	
	Hepatitis	11 (22.4)	7 (22.6)	4 (22.2)	
	Alcoholism	10 (20.4)	8 (25.8)	2 (11.1)	
	Cryptogenic	7 (14.3)	5 (16.1)	2 (11.1)	
	Others	9 (18.4)			
**Child-Pugh** *					0.34
	A	12 (24.5)	7 (22.6)	5 (27.8)	
	B	13 (26.5)	8 (25.8)	5 (27.8)	
	C	18 (36.7)	13 (41.9)	5 (27.8)	
	MELD	20 (18.3-24)	20 (18-23)	20 (19.7-24)	0.73
	Baseline SCr	0.91 (0.69-1.20)	0.89 (0.66-0.99)	0.98 (0.74-1.35)	0.188
**Donor data**					
	Age in years	338 (24-51)	41 (23-53)	31.5 (26.7-47.2)	0.67
	Sex, male	33 (67.3)	22 (71)	11 (61)	0.53
	Vasopressor use	29 (59)	16 (51.6)	13 (72.2)	0.23
**Surgical/perioperative data**					
	Surgical time	407 (309-549)	375 (300-467)	453 (331-633)	0.07
	Cold ischemia time	466 (405-633)	473 (375-620)	460 (412-661)	0.3
	Vasopressor use	32 (65.3)	19 (61.3)	13 (72.2)	0.54
	Blood transfusion	27 (55.1)	16 (51.6)	11 (61.1)	0.28
**Postoperative data**					
	Reintervention	12 (24.5)	4 (12.9)	8 (44.4)	0.01
	Vasopressor requirement	36 (73.5)	19 (61.3)	17 (94.4)	0.01
	Albumin use for more than 24 hours	20 (40.8)	11 (35.5)	9 (50)	0.37
	Hemoglobin 6 hours		9.5 (8.5-11.9)	8.5 (7.2-10.4)	0.19
	Hemoglobin 24 hours#		8.5 (6.8-11.0)	7.2 (6.7-9.2)	0.969
	Creatinine 6 hours	1.04 (0.77-1.67)	0.95 (0.71-1.16)	1.5 (1.0-2.39)	0.003
	Lactate within 6 hours	2.8 (1.9-4.3)	2.38 (1.78-3.22)	3.31 (2.67-7.28)	0.01
	Lactate 24 hours	2.0 (1.44-3.0)	1.82 (1.25-2.27)	4.0 (1.84-6.0)	< 0.001
	Transfusion in ICU	28 (57.1)	13 (41.9)	15 (83.3)	0.01

Data are presented as n (%) or median (Q1-Q3).

* Referring to 45 patients

# Referring to 47 patients; Q1-Q3 = interquartile range; AKI = acute kidney injury; SAH = systemic arterial hypertension; DM = diabetes mellitus; CKD = chronic kidney disease; HCC = hepatocellular carcinoma; MELD = Model of End-Stage Liver Disease; ICU = intensive care unit; SCr = serum creatinine.

No preoperative factor assessed in this study was associated with KDIGO 2/3 development. Forty-one patients (83.6%) developed AKI during the follow-up period; 23 (46.9%) with KDIGO 1, two (4.1%) with KDIGO 2 and 16 (32.7%) with KDIGO 3.

### Factors associated with AKI during ICU stay

Vasopressor requirement occurred more frequently in patients with AKI KDIGO 2 or 3 than those with non-AKI/KDIGO 1 (17 [94.4%] versus 19 [61.3%] patients; P = 0.01). Moreover, the former group received more blood transfusion during the first week of ICU stay (15 [83.3%] versus 13 [41.9%] patients; P < 0.01) and required surgical reintervention more often (8 [44.4%] versus 4 [12.9%] patients; P = 0.01). In addition, the AKI KDIGO 2/3 group had higher creatinine levels (1.5 [1.0-2.39] versus 0.95 [0.71-1.16] mg/dl; P = 0.003) and higher arterial lactate levels within the first six hours after admission (3.31 [2.67-7.28] versus 2.38 [1.78-3.22] mmol/l; P = 0.01) than their counterparts. There was no difference between the two groups with regard to other baseline characteristics (Table 1).

In a multivariate analysis, ICU blood transfusion requirement (odds ratio, OR: 8.4; 95% confidence interval, CI: 1.68-41.824; P = 0.009) and higher arterial lactate level measured within the first six hours after ICU admission (OR: 1.32; 95% CI: 1.021-1.717; P < 0.05) were independently associated with development of AKI KDIGO 2 or 3 (Table 2).

**Table 2:** Univariate and multivariate analysis on factors associated with development of acute kidney injury KDIGO 2 or 3

Characteristics	Univariate		Multivariate	
	P - value	OR	95% CI	P-value
Vasopressor requirement	0.01			
Hemoglobin 6 hours	0.19			
Lactate 24 hours	< 0.001			
Reintervention	0.01			
Transfusion in ICU	0.01	8.400	1.68-41.824	0.009
Lactate within 6 hours	0.01	1.324	1.021-1.717	0.034

Data are presented as n (%) or median and interquartile range (Q1-Q3).

ICU = intensive care unit; OR = odds ratio; CI = confidence interval.

### AKI and patient outcomes

The in-hospital mortality rate was 22.4% (Table 3). Patients with AKI KDIGO 2 or 3 throughout the first seven days of follow-up underwent RRT more frequently (13 [72.2%] versus 1 [3.2%] patients; P < 0.01), had a longer hospital stay in days (20 [7.2-31.7] versus 15 [11-29] days; P < 0.01), higher in-hospital mortality (8 [44.4%] versus 2 [6.5%] patients; P < 0.01) and a higher mortality rate after one year of follow-up (8 [44.4%] versus 3 [9.7%] patients; P < 0.01), compared with the non-AKI or KDIGO 1 subgroup (Table 3).

**Table 3:** Outcomes according to development of acute kidney injury (KDIGO 0/1 and KDIGO 2/3)

Outcomes	All	KDIGO 0/1	KDIGO 2/3	P-value
	(n = 49)	(n = 31)	(n = 18)	
RRT in ICU	14 (28.6)	1 (3.2)	13 (72.2)	< 0.01
Days in ICU	6 (3-9)	5 (3-8)	7.5 (3-12.5)	0.59
Days in hospital	18 (11-30)	15 (11-29)	20 (7.2-31.7)	0.001
In-hospital mortality	10 (20.4)	2 (6.5)	8 (44.4)	< 0.01
One-year mortality	11 (22.4)	3 (9.7)	8 (44.4)	0.01

Data are presented as n (%) or median and interquartile range (Q1-Q3).

RRT = renal replacement therapy; ICU = intensive care unit.

## DISCUSSION

In this study, occurrence of AKI KDIGO 2 or 3 during the postoperative period following liver transplantation was common (36.7%) and was independently associated with a requirement for blood transfusion during the ICU stay and with higher levels of lactate during the first six hours after ICU admission. Additionally, our exploratory data suggested that patients who developed AKI at these stages had worse outcomes, such as longer ICU and hospital stay and higher short and long-term mortality rates.

Previous studies have suggested that pre-transplantation renal impairment, measured through creatinine levels, plays an essential role in the development of postoperative AKI.^21–23^ It is well known that in cirrhotic patients, serum creatinine levels can be falsely low due to malnutrition, reduced muscle mass or decreased creatinine biosynthesis.^24^ In order to better identify occurrences of AKI, we did not include patients with dialytic chronic end-stage renal failure and those undergoing double renal-hepatic transplantation in our study. Through this strategy, we intentionally selected individuals with serum creatinine values within the normal range, which thus precluded investigation of the role of pre-transplantation altered creatinine levels for predicting AKI in the postoperative period.

A requirement for vasoactive drugs during the intraoperative period has previously been correlated with AKI development among liver transplantation patients. Karapanagiotou et al. showed that use of vasopressors during the intraoperative period was associated with higher rates of AKI, assessed through the RIFLE and AKIN scores.^25^ Other authors found similar results using the KDIGO score.^2,22,26^ However, even though the majority of the data speaks in favor of the existence of an association between intraoperative use of vasopressors and AKI, this issue remains a matter of debate.^27^ For instance, some authors have suggested that norepinephrine might protect against AKI development, through improving renal blood flow.^28^ In our study, a requirement for vasopressors during the ICU stay but not during the preoperative period was shown to be associated with AKI. Similarly, Zhou et al. showed that the need for vasopressors to maintain blood pressure at a minimum of 65 mmHg (mercury millimeters) during the postoperative period resulted in a fivefold increase in AKI incidence.^12^

Knowledge of postoperative risk factors associated with AKI in liver transplantation patients is scarce: this has mostly been studied in the pre and intraoperative periods.^23,27,28–30^ It is currently believed that bleeding that leads to transfusion requirements during the surgical procedure is associated with AKI, especially a need for red blood cells and cryoprecipitate.^6,22,30^ In our study, the need for blood transfusion at any time during the ICU stay was associated with an odds ratio eight times higher for the development of AKI KDIGO 2 or 3.

Erdost et al. evaluated the three surgical periods among liver transplantation patients and found that only in the intraoperative period was blood transfusion associated with AKI development.^30^ Zongyi et al. also evaluated the three surgical periods and identified that transfusion of red blood cells and fresh frozen plasma in the intraoperative period was associated with postoperative AKI development in liver transplantation patients.^29^ These authors did not find that the need for transfusion in the postoperative period was a risk factor associated with development of AKI, but they noted that there was an association between postoperative intraperitoneal hemorrhage and AKI.^29^ We hypothesize that the need for blood transfusion is an indirect marker of the severity of patients’ condition, such that it is more frequent among patients with postoperative complications and among those who need surgical reintervention.

We showed that higher serum lactate levels within the first six hours after ICU admission were independently associated with development of AKI KDIGO 2/3. In agreement with our findings, Jipa et al. showed that lactate levels greater than 1.5 mmol/l were related to postoperative complications in liver transplantation patients, including AKI, need for surgical reintervention and graft dysfunction.^31^ Furthermore, Rueggeberg et al. observed that hyperlactatemia observed at the end of surgery was associated with increased intraoperative bleeding, longer ICU stay and increased mortality.^31,32^ Given that lactate is primarily metabolized in the liver, higher levels of this molecule are observed in cases of liver dysfunction, regardless of the etiology. However, in cases of ischemic injuries, such as thrombosis of the vascular graft in liver transplantation, a sharp increase in lactate levels might be observed. In our study, patients with renal impairment showed higher lactate levels than those without kidney injury, thus suggesting that the former group probably had some liver harm or hypoperfusion.

Regarding the consequences of AKI during the postoperative period following liver transplantation, our study showed that patients with KDIGO 2 or 3 required renal replacement therapy (RRT) more often and had longer hospital stays and higher in-hospital mortality, compared with the KDIGO 1 or non-AKI patients. Similarly, Lima et al. found that severe AKI (KDIGO 2 or 3) was associated with RRT therapy, longer hospital and ICU stays and higher mortality over a 60-day period.^9^ These are somewhat expected findings, since AKI probably serves as a marker for other types of organ dysfunction and for clinical severity in general.

This study had limitations that need to be considered. Firstly, because of the retrospective design, we were unable to assess diuresis in our definition of AKI, quantify vasopressor use or analyze the use of each vasopressor separately. Also, we did not evaluate early calcineurin therapy in the postoperative period following liver transplantation (e.g. tacrolimus serum levels). Secondly, this was a single-center study, with inclusion of a small number of patients, thus limiting the power of our statistical inferences. Thirdly, we were unable to characterize the severity of illness of the patients included using a severity score (e.g. Acute Physiology and Chronic Health Evaluation II [APACHE II] or Simplified Acute Physiology Score 3 [SAPS 3]), which precluded any adjustment of our associative analyses for this relevant parameter.

## CONCLUSION

This study showed that the need for blood transfusion during ICU stay and hyperlactatemia at ICU admission are independently associated with development of AKI KDIGO stages 2 or 3. Furthermore, our findings suggest that AKI patients require RRT more often, have a longer hospital stay and might have higher short and long-term mortality. Prospective studies are needed in order to better identify early factors associated with acute renal function loss during the immediate postoperative period following liver transplantation, thereby enabling more assertive and prompter interventions that might result in better clinical outcomes.
